# Editorial: Applications of bioinformatics, machine learning and risk analysis for microbial food safety

**DOI:** 10.3389/fmicb.2024.1414261

**Published:** 2024-05-03

**Authors:** Bing Niu

**Affiliations:** School of Life Science, Shanghai University, Shanghai, China

**Keywords:** foodborne pathogens, food safety, bioinformatics, machine learning, simulation, risk analysis

Food safety remains a global concern as foodborne illnesses persist, stemming from various microorganisms like norovirus, hepatitis A, Salmonella, *E. coli*, and mycotoxin-producing fungi. The advent of sequencing, bioinformatics, and big data technologies opens new avenues in addressing food safety challenges. Innovative strategies, driven by bioinformatics, machine learning, and risk analysis, are enhancing the detection, diagnosis, prediction, and prevention of foodborne disease outbreaks. These advancements offer valuable recommendations to mitigate microbial food safety hazards.

The Research Topic features four noteworthy papers focusing on machine learning methods and bioinformatics techniques. These papers explore aflatoxins prediction, non-targeted metabolomics of moldy wheat, eae-positive Shiga toxin-producing *Escherichia coli*, and microbial communities in fresh produce. Each contributes to the evolving landscape of computational technologies in ensuring food safety on an international scale.

Aflatoxin, produced by fungi in corn, poses health risks for humans and livestock. Branstad-Spates et al. focused on developing a Gradient Boosting Machine (GBM) learning model to predict aflatoxin (AFL) contamination in Iowa corn, aiming to enhance food and feed safety. T The model incorporates historical corn contamination, meteorological, satellite, and soil data from Iowa, the leading corn-producing state in the US. Assessing two risk thresholds (20- and 5-ppb) with a 90–10% training-to-testing ratio for 2010, 2011, 2012, and 2021, independently validated in 2020, the GBM model achieved 96.77% accuracy for a 20-ppb threshold and 90.32% for a 5-ppb threshold, despite limited sensitivity to high AFL contamination events. Influential factors identified include the August satellite-derived vegetative index, aflatoxin risk indices in May and July, latitude, and soil-saturated hydraulic conductivity. Developing annual AFL predictive models proves practical for grain handling, emphasizing proactive measures critical for hazard management and optimizing the nation's corn crop safety and efficiency (Branstad-Spates et al.).

Gao et al. addressed the challenge of ensuring wheat safety, a staple for global populations, focusing on the pervasive issue of mildew affecting wheat quality during its growth, production, and storage. Rapidly identifying moldy wheat is complex due to intricate microbial metabolites. Using ultraperformance liquid chromatography—quadrupole time-of-flight mass spectrometry (UPLC-QTOF-MS) and chemometrics, the study established a non-targeted PCA model with a compounds database of authentic wheat samples. This model efficiently discriminates between moldy and normal wheat. Employing orthogonal projection to latent structures-discrimination analysis (OPLS-DA) with optimized parameters, the study accurately identified moldy wheat, even at 5% (w/w) adulteration levels. Unique biomarkers for moldy wheat were extracted, demonstrating the efficacy of combining chemical information with the PCA model. This research introduces a powerful method for screening wheat safety, contributing to the wellbeing of individuals relying on wheat as a dietary staple (Gao et al.).

Vorimore et al. sought to create a precise model for predicting highly pathogenic Shiga toxin-producing *Escherichia coli* (STEC) in complex *E. coli* samples. Utilizing genome-wide machine learning, they considered *E. coli*'s genomic diversity, stratifying STEC and *E. coli* pathogroups based on serotype and virulence factors. The focus was on identifying biomarkers for characterizing eae-positive STEC associated with severe human conditions. With a dataset of 1,493 *E. coli* genome sequences and 1,178 Coding Sequences (CDS), eight classification algorithms selected six key CDS. Machine learning models, tuned and validated, demonstrated the capability to identify enterohemorrhagic *E. coli* (EHEC) using only these six genes in complex samples like milk metagenomes. These biomarkers show potential for clear EHEC characterization in diverse *E. coli* strain mixtures and raw milk metagenomes, offering insights into food safety and public health (Vorimore et al.).

Townsend et al. investigated microbial communities in 18 food-handling distribution centers (DCs) across the United States using 16S amplicon sequencing on 317 environmental surface swabs. Significant diversity variations were observed among individual DCs, with top genera including Carnobacterium_A, Psychrobacter, Pseudomonas_E, Leaf454, and Staphylococcus. Four samples containing Listeria amplicon sequence variants correlated with positive Listeria microbiological samples. Cold-tolerant bacteria were prevalent in DC environmental samples. Differential abundance analysis revealed higher levels of Carnobacterium_A, Psychrobacter, and Pseudomonas_E in Listeria-positive samples. Microbiome composition varied significantly based on DC, season, and general sampling location. This research highlights potential pathogen presence and variations in microbial dynamics within food-related DCs, contributing to our understanding of microbial ecology in these environments (Townsend et al.).

## Author contributions

BN: Writing—review & editing, Writing—original draft.

